# Detection of *Burkholderia pseudomallei* in Sputum using Selective Enrichment Broth and Ashdown’s Medium at Kampong Cham Provincial Hospital, Cambodia

**DOI:** 10.12688/f1000research.5935.2

**Published:** 2015-05-15

**Authors:** Somary Nhem, Joanne Letchford, Chea Meas, Sovanndeth Thann, James C. McLaughlin, Ellen Jo Baron, T. Eoin West

**Affiliations:** 1 Kampong Cham Provincial Hospital, Kampong Cham, Cambodia; 2 Diagnostic Microbiology Development Program, Phnom Penh, Cambodia; 3 Department of Pathology, Stanford University School of Medicine, Palo Alto, CA 94304, USA; 4 International Respiratory and Severe Illness Center, Division of Pulmonary and Critical Care Medicine, Harborview Medical Center, University of Washington, Seattle, WA 98104, USA

**Keywords:** Burkholderia pseudomallei, melioidosis, pneumonia, diagnostic microbiology, lung, sputum, Cambodia

## Abstract

Melioidosis, infection caused by *Burkholderia pseudomallei*, is increasingly reported in Cambodia. We hypothesized that implementation of an enhanced sputum testing protocol in a provincial hospital diagnostic microbiology laboratory would increase detection of *B. pseudomallei*. We tested 241 sputum specimens that were deemed acceptable for culture, comparing culture in selective enrichment broth followed by sub-culture on Ashdown’s medium to standard culture methods. Two specimens (0.8%) were positive for *B. pseudomallei* using the enhanced protocol whereas one specimen (0.4%) was positive using standard methods. Given the low numbers of positive specimens, we could not conclusively determine the utility of the enhanced sputum testing protocol. However, the ramifications of identification of *B. pseudomallei* are substantial, and the benefit of the enhanced testing protocol may be more apparent in patients selected based on risk factors and clinical presentation. Promoting clinician awareness of the infection and encouraging utilization of diagnostic microbiology services are also likely to be important factors in facilitating identification of melioidosis.

## Introduction

Melioidosis, infection with the Gram negative bacterium *Burkholderia pseudomallei*, has increasingly been described in Cambodia in recent years ^1– 6^. Infection is caused by inoculation, inhalation, or ingestion of *B. pseudomallei*, an environmental saprophyte ^7^. In one single center Cambodian study, melioidosis (defined as growth of *B. pseudomallei* in any clinical specimen) was lethal in more than half of cases, many of whom had received inappropriate initial antibiotic therapy ^2^. The clinical presentation of melioidosis is notoriously variable, but pneumonia and sepsis occur commonly ^7^. Sub-acute and chronic forms of melioidosis may be confused with tuberculosis and it is important to distinguish these diseases ^8^. *B. pseudomallei* is resistant to penicillin, ampicillin, first- and second-generation cephalosporins and aminoglycosides ^7^. Use of quinolones or third-generation cephalosporins to treat melioidosis is associated with poorer outcomes ^9– 11^. Thus, many of the first line antimicrobial therapies administered for pneumonia and sepsis do not adequately treat melioidosis. The treatment course for melioidosis is prolonged, requiring initial intensive therapy for at least 10–14 days with intravenous ceftazidime, imipenem, or meropenem, followed by 3–6 months of oral eradication therapy with trimethoprim-sulfamethoxazole ^7^. Moreover, ceftazidime and carbapenems are relatively expensive and may not be universally accessible in Cambodia. Therefore, rapid diagnosis of the infection is essential to guide clinical management. Culture is the gold standard diagnostic test for melioidosis and recovery of *B. pseudomallei* from any sample constitutes infection. In pulmonary melioidosis, culture of sputum may provide critical diagnostic information. However, in culturing *B. pseudomallei* from non-sterile samples such as sputum, it is important to minimize overgrowth by commensal or contaminating bacteria by using selective media. In this report, we present our experience implementing an enhanced sputum testing protocol in a Cambodian provincial hospital diagnostic microbiology laboratory to increase detection of *B. pseudomallei*.

Kampong Cham Provincial Hospital is a 260 bed referral hospital approximately 120 kilometers by road northeast of Phnom Penh in the city of Kampong Cham. The province of Kampong Cham is home to about 1.75 million people; major industries are agriculture and agri-business [ http://www.cambodiainvestment.gov.kh/kampong-cham-province.html, accessed 05/06/15]. The diagnostic microbiology laboratory at the hospital began offering culture and antibiotic susceptibility testing in 2009. Several cases of *B. pseudomallei* have been isolated at the laboratory since then. Given resource limitations, standard media used to culture sputum specimens in this laboratory are blood, chocolate, and MacConkey agars. In northeast Thailand, where melioidosis is highly endemic ^7^, the use of selective enrichment broth and Ashdown’s medium has enhanced the detection of *B. pseudomallei* from sputum ^12– 14^. We hypothesized that the use of selective enrichment broth and Ashdown’s medium at Kampong Cham Provincial Hospital would similarly increase the identification of cases of pulmonary melioidosis.

## Methods

To compare the use of selective enrichment broth and Ashdown’s medium to standard sputum culture at Kampong Cham Provincial Hospital, we performed specific testing for *B. pseudomallei* on all sputum specimens submitted to the laboratory and accepted for bacterial culture between March 25 and September 30, 2013, following a previously described protocol ^12, 14^. We chose these dates to include much of the rainy season, when melioidosis is diagnosed more frequently ^2^. No data were available on the subjects who provided the sputum specimens although most specimens were submitted from the hospital’s tuberculosis screening area or ward. Specimens accepted for culture had fewer than 10 epithelial cells per low power field or moderate to high numbers of polymorphonuclear cells on Gram stain. Specimens were inoculated into selective enrichment broth containing Tryptic Soy Broth 10g, glycerol 40ml, crystal violet 0.1% 5ml, and colistin 50mg per liter of distilled water for two days at 37°C in aerobic conditions. Specimens were then sub-cultured onto Ashdown’s medium (Tryptic Soy Broth 10g, agar 15g, glycerol 40ml, crystal violet 0.1% 5ml, neutral red 1% 5ml, and gentamicin 4mg per liter of distilled water) and incubated for four days at 37°C in aerobic conditions. Colonies that grew were tested using the oxidase test; oxidase-positive colonies were tested using a highly sensitive and specific monoclonal antibody-based latex agglutination test for *B. pseudomallei* ^15, 16^. In parallel, as per routine laboratory practice, all sputum specimens were also cultured onto sheep blood, chocolate, and MacConkey agars.

## Results

Two hundred and forty one sputum specimens deemed acceptable for culture were received by the laboratory during the study period and tested using the enhanced protocol. *B. pseudomallei* was isolated from one specimen (0.4%) using standard media and from two specimens (0.8%) using the enhanced protocol. The single specimen positive using standard media was also positive using the enhanced protocol; the enhanced technique therefore detected one additional isolate of *B. pseudomallei* compared to the standard protocol. In this case, *Klebsiella pneumoniae* grew on standard media, raising the possibility that overgrowth of *K. pneumoniae* precluded detection of *B. pseudomallei* in the absence of selective enrichment broth. Among the 241 sputum specimens collected during the study period, *B. pseudomallei* accounted for two (1.6%) of the 122 that were culture positive. Other bacteria isolated from the sputum samples during the study period were *K. pneumoniae* (54), *Pseudomonas* species (37), *Enterobacter* species (7), *Acinetobacter* species (5), *Staphylococcus aureus* (4), *Stenotrophomonas maltophilia* (3), *Streptococcus pneumoniae* (3), *Haemophilus influenzae* (3), *Escherichia coli* (3), *K. ozaenae* (3), unknown Enterobacteriaceae (2), *Vibrio alginolyticus* (1), and a non-fermenting Gram negative bacillus (1). On standard media, three other specimens were positive for *B. pseudomallei* during the study period: a pleural fluid specimen from one patient with a *B. pseudomallei*-positive sputum culture, and pus from two other individuals.

Detection of *B. pseudomallei* in Sputum using Selective Enrichment Broth and Ashdown’s Medium Compared to Standard Culture Specimens (Column A) were inoculated into selective enrichment broth before sub-culture onto Ashdown’s medium (Column B). Colonies that grew were tested using the oxidase test (Column C); oxidase-positive colonies were tested using a monoclonal antibody-based latex agglutination test for *B. pseudomallei* (Column D). The final results of this testing method are given in Column E. All sputum specimens were also cultured onto sheep blood, chocolate, and MacConkey agars. The results of standard culture are given in Column F. NRF: normal respiratory flora.

## Conclusions/Discussion

These data confirm that respiratory melioidosis is detected in patients presenting to Kampong Cham Provincial Hospital although the overall number of sputum specimens positive for *B. pseudomallei* was low. Our rate of detection of *B. pseudomallei* in sputum samples submitted to the laboratory is similar to a previously published study evaluating the etiology of clinical respiratory infection at this hospital ^3^. This study of patients with acute lower respiratory tract infection identified melioidosis in four of 422 (0.9%) patients from the hospital. The diagnosis was determined by culture of *B. pseudomallei* from sputum or blood at an off-site laboratory.

In light of the small number of specimens positive for *B. pseudomallei* we could not definitively ascertain an advantage to the routine use of the enhanced sputum testing protocol. However, the ramifications of identification of *B. pseudomallei* are substantial. Although ceftazidime and imipenem may not be available in Cambodian hospital pharmacies and cost prevents the outside purchase by most patients, mortality from melioidosis is greatly diminished when appropriate antibiotic therapy is instituted ^17^. Others have reported a benefit of a similar diagnostic testing strategy. For example, the use of selective enrichment broth and *B. pseudomallei* selective agar to test 154 respiratory samples in a referral center in Kuala Lumpur identified three cases of *B. pseudomallei* that were not detected using routine media ^18^.

Our results should be interpreted considering the following limitations. First, as with all tests, the selection of patients and indications for sampling are highly likely to influence the results. We unfortunately do not have those important data. Second, the cost of sputum culture may have been prohibitive for many patients, further skewing the population sampled. Third, a relatively small number of sputum specimens were submitted to the laboratory during the six month period. As the hospital microbiology laboratory has only been operational since 2009, this may reflect the established clinical practice of not relying on microbiology data to make treatment decisions. Thus, a number of factors may contribute to the low rates of detection of *B. pseudomallei* in sputum samples tested in this study; future studies underway aim to address these issues.

We further observed that the majority of sputum specimens came from patients presenting to the hospital’s tuberculosis screening area and ward. This suggests that in many cases clinicians may have been considering the diagnosis of tuberculosis. Melioidosis and tuberculosis may be difficult to distinguish clinically ^8^ and both infections should be considered in patients with chronic symptoms of respiratory infection in Cambodia. However, most respiratory melioidosis is associated with symptoms for less than two months and the mean incubation period in acute melioidosis is nine days ^19, 20^. We speculate that sputum from melioidosis patients presenting elsewhere in the hospital with acute clinical syndromes of pneumonia and sepsis may not have been submitted for culture. This may have reduced our rate of detection of melioidosis in this study. Furthermore, this highlights the importance of informing clinicians of the utility of diagnostic microbiology testing, preferably before initiation of treatment.

Several other techniques should also be considered in the diagnosis of pulmonary melioidosis. Many cases of pulmonary melioidosis have concurrent bacteremia ^19^ and *B. pseudomallei* bacteremia is reported in Cambodia. In a recent study, *B. pseudomallei* accounted for 12.6% of clinically significant positive blood cultures collected from adults between July 2007 and December 2010 at the Sihanouk Hospital Centre of HOPE, Phnom Penh ^21^. From January to December 2013, *B. pseudomallei* was isolated in 18 of 2,230 (0.8%) blood cultures submitted to microbiology laboratories at five Cambodian government hospitals, with the majority of *B. pseudomallei* cultures (15/434, 3.5%) occurring at Takeo Provincial Hospital (unpublished data). Throat swabs cultured in selective enrichment broth, while insensitive, are easily obtained and highly specific for the diagnosis of melioidosis ^22, 23^. Thus, routinely culturing blood and throat swabs in patients with pneumonia and sepsis may enhance the diagnosis of respiratory melioidosis in Cambodia.

In conclusion, these data confirm that *B. pseudomallei* is a respiratory pathogen in Kampong Cham province but show that it is rarely detected in sputum samples submitted to the diagnostic microbiology laboratory at Kampong Cham Provincial Hospital. As a result, our study cannot conclusively determine the utility of an enhanced sputum testing protocol at this site. The benefit of the enhanced testing protocol may be more apparent in patients selected based on risk factors and clinical presentation. Future studies will investigate this possibility. In addition, given the severity of melioidosis and importance of identifying the infection to guide treatment, several other factors should be considered in order to increase detection of the disease. In particular, clinician awareness of the diverse presentations of melioidosis and routine utilization of diagnostic microbiology services for patients presenting with pneumonia and sepsis should be promoted.

## Data availability

*F1000Research*: Dataset 1. Detection of *B. pseudomallei* in Sputum using Selective Enrichment Broth and Ashdown’s Medium Compared to Standard Culture, 10.5256/f1000research.5935.d40423 ^24^
