# Supplementary material for: Detection of Burkholderia pseudomallei in Sputum using Selective Enrichment Broth and Ashdown’s Medium at Kampong Cham Provincial Hospital, Cambodia
Source: F1000Res. 2015 May 15;3:302. Originally published 2014 Dec 11. [Version 2] doi: 10.12688/f1000research.5935.2 (PMC4329667; doi:10.12688/f1000research.5935.2)
Supplement: Peer review history

# Peer review: Referee response for version 1

**DOI:** 10.5256/f1000research.6343.r7217

**Published:** 2015-02-06

**Authors:** Jan Jacobs

**Affiliations:** 1 Department of Clinical Sciences, Institute of Tropical Medicine, Antwerp, Belgium

This is a well-designed and clearly written paper about describing the incremental yield of selective culture for Burkholderia pseudomallei (BPM) in sputum samples routinely submitted for culture in a Provincial hospital in Cambodia, where melioidosis is endemic.

I have the following comments:

1. Most readers will not be familiar with melioidosis. It may be useful to state more explicitly in the Introduction that culture for the time being is the only reliable way for diagnosis of melioidosis and that recovery of BPM from any sample and in any quantity always means infection: this is especially important as for most other bacteria grown from non-sterile samples (as respiratory secretions), colonization and oral flora contamination must be excluded, which may be done by, among other criteria, based on density of growth. Second, based on this observation, selective media have been recommended as part of routine sputum culture in endemic areas.
2. The main weakness of this study is that there are no data about indications of sampling and selection of patients and from the Discussion can be learned that probably no well-defined indications have been used. So it is not clear whether all patients were suspected of lower respiratory tract infection and acutely ill, or if  they selected, for instance, based upon initial treatment success and ability to pay for the culture etc.. The authors should emphasize this non-selection somewhat more, and, for instance, display data or at least an idea about the proportion of patients cultured in relation to the total (estimated) numbers of eligible patients with acute respiratory tract infections. Likewise, some data about the patients’ profile (age (median/range or IQR) and gender would be of interest, as well as the distribution between hospitalized/outpatients -  the spectrum of organisms cultured as mentioned in the Results suggests a high proportion of hospitalized patients.
3. In line with the above, I think the authors should reconsider amendingtheir conclusion: they are right by stating that selective enrichment culture media would be more rewarding in settings with high(er) incidence of melioidosis, but there is for the time being no sound evidence about the to assume that the incidence of melioidosis in their place will be lower than, for instance, in Thailand. Moreover, and as the authors state in the Discussion above, sputum and other microbiological cultures are probably not yet exploited to the best extent by the clinicians and probably also cost-prohibitive for many patients, reason why identification and selection of at-risk patients and pre-test probability may have been more important than overall incidence of melioidosis (as the authors state in the Discussion). In other words, if the enhanced testing protocol would have applied on for instance, middle-aged patients with diabetes presenting with overt signs of pneumonia/sepsis, incremental yield and cost-effectiveness may be much higher. So I suggest to consider rephrasing “the benefit …may be more apparent … in patients selected upon risk factors and clinical presentation…” Likewise, the Abstract (final sentence) may be rephrased to focus more on selected indications for sputum sampling.
4. The authors may add some information about the referral population of Kampong Cham hospital: rural, agriculture...

I have read this submission. I believe that I have an appropriate level of expertise to confirm that it is of an acceptable scientific standard.

# Response

**Published:** 2015-05-07

**Authors:** T Eoin West

**Affiliations:** Division of Pulmonary & Critical Care Medicine, University of Washington, USA

Thank you for this constructive review.

1.  We agree and have modified the introduction to clarify these points as suggested.
2. This is indeed a shortcoming of this study; unfortunately we do not have any data on the patients who provided the sputum samples tested for these patients. Sputum samples are submitted to the laboratory from both clinic and hospitalized patients. Our future studies aim to investigate patient characteristics and indications for sampling.
3. We agree with these very helpful comments, and have modified the abstract and discussion as suggested.
4. We have now added this information as suggested.

# Peer review: Referee response for version 1

**DOI:** 10.5256/f1000research.6343.r7216

**Published:** 2015-01-20

**Authors:** David A. B. Dance

**Affiliations:** 1 Centre for Tropical Medicine and Global Health, Nuffield Department of Clinical Medicine, Churchill Hospital, University of Oxford, Oxford, UK

This paper describes an evaluation of the use of a selective enrichment method to detect *Burkholderia pseudomallei *in sputum samples in a provincial hospital in Cambodia. The additional yield compared with conventional cultures was relatively low (only one of 241 samples tested). 

The paper is well written and presents the results clearly. It would have been improved by the inclusion of an assessment of the additional costs of using selective culture. Clearly the benefits in this hospital are relatively small (although potentially life-saving for the one additional patient with melioidosis who was detected), but cannot be extrapolated to other areas because the distribution of melioidosis may be remarkably focal (for example in Thailand the incidence is very low in the central region but high in the northeast). It is also unclear why a selective enrichment technique was used as opposed to direct culture onto solid media, since enrichment culture was not shown significantly to increase the *B. pseudomallei* isolation rate from sputum compared with plating directly onto Ashdown's medium in a previous study ( Wuthiekanun *et al.*,1990). It might also have been possible to have been more selective about the samples that were cultured and to have analysed patient characteristics in greater detail, although the majority of samples were thought to have been from patients suspected of having TB, a group in whom it has previously been suggested that testing for melioidosis (using a similar enrichment technique) might be warranted ( Suntornsut *et al*., 2013).

Although this is a relatively small scale study with relatively unremarkable results, it warrants being in the public domain. Laboratories in melioidosis-endemic areas should still be encouraged to consider looking for *B. pseudomallei *in appropriate samples and should conduct their own assessment of the costs and benefits of doing so based on local epidemiology.

I have read this submission. I believe that I have an appropriate level of expertise to confirm that it is of an acceptable scientific standard.

# Response

**Published:** 2015-05-07

**Authors:** T Eoin West

**Affiliations:** Division of Pulmonary & Critical Care Medicine, University of Washington, USA

Thank you for the helpful comments.

We opted not to perform cost-benefit analyses in light of our small numbers of positive samples and wide confidence intervals around point estimates. We agree that these calculations are important to perform in the future in larger studies.

We chose to use selective broth based on Wuthiekanun V et al, Am J Trop Med Hyg 77(5), 2007, 812-13. This study reported that “a total of 94 of 120 (78%) respiratory secretions were positive for B. pseudomallei, of which 33 (35%) were positive from enrichment broth alone.”

Unfortunately we do not have any data on patient characteristics. Our future studies aim to investigate patient characteristics and indications for sampling.
